# Hydrogel scaffolds in bone regeneration: Their promising roles in angiogenesis

**DOI:** 10.3389/fphar.2023.1050954

**Published:** 2023-02-13

**Authors:** Jun Liu, Lili Yang, Kexin Liu, Feng Gao

**Affiliations:** ^1^ Department of Hand Surgery, The Second Hospital of Jilin University, Changchun, China; ^2^ Department of Spinal Surgery, The Second Hospital of Jilin University, Changchun, China; ^3^ Department of Gastrointestinal Colorectal Surgery, China-Japan Union Hospital of Jilin University, Changchun, China; ^4^ Department of Orthopedics, The Second Hospital of Jilin University, Changchun, China

**Keywords:** bone tissue engineering, scaffold, biomaterial, hydrogel, angiogenesis

## Abstract

Bone tissue engineering (BTE) has become a hopeful potential treatment strategy for large bone defects, including bone tumors, trauma, and extensive fractures, where the self-healing property of bone cannot repair the defect. Bone tissue engineering is composed of three main elements: progenitor/stem cells, scaffold, and growth factors/biochemical cues. Among the various biomaterial scaffolds, hydrogels are broadly used in bone tissue engineering owing to their biocompatibility, controllable mechanical characteristics, osteoconductive, and osteoinductive properties. During bone tissue engineering, angiogenesis plays a central role in the failure or success of bone reconstruction *via* discarding wastes and providing oxygen, minerals, nutrients, and growth factors to the injured microenvironment. This review presents an overview of bone tissue engineering and its requirements, hydrogel structure and characterization, the applications of hydrogels in bone regeneration, and the promising roles of hydrogels in bone angiogenesis during bone tissue engineering.

## 1 Introduction

Bone is the largest tissue in the human body with several pivotal physiological functions, such as detoxification and maintaining body shape, as well as its considerable role as an endocrine organ in keeping and releasing mineral substances (DiGirolamo et al., 2012; Xue et al., 2021). Bone defects, including infection, trauma, and tumor, could impair its functions. Although the self-regenerative property of bone tissue repairs the defects, bone regeneration cannot heal when defects exceed critical size (ASTM, 2014). As a gold standard, autografts are used to repair critical-sized bone defects in orthopedics due to their excellent osseointegration, osteoconductivity, and osteoinductivity properties (Zhu et al., 2021). Despite promising outcomes, autografts also have some critical limitations, including disease transfer, inflammation, and lack of donor bone transplantation (Younger and Chapman, 1989; St John et al., 2003). Additionally, autografts have a heavy economic burden on healthcare systems. It has been estimated that the bone graft substitutes market will reach 5 × 10^9^ $ in 2025 (Lobb et al., 2019). In Germany, it has been reported that autologous and allogenic bone graft percentages changed up to −14.3% and +74.1%, respectively, between 2008–2018, whereas the using biomaterials increased up to +134.4% (Rupp et al., 2022). Bone tissue engineering (BTE) has been developed to address bone failures with new prospects.

Several biomaterials, as scaffolds, have been designed and tested in BTE owing to their unique characteristics. Polylactic acid (PLA), polyglycolic acid (PGA), poly (lactic-co-glycolic acid) (PLGA), poly ɛ-caprolactone (PCL), polyethylene glycol (PEG), polybutylene terephthalate (PBT), polyethylene terephthalate (PET), polyvinyl alcohol (PVA), polypropylene fumarate (PPF), and polyacrylic acid (PAA) have been approved by FDA for BTE as polymeric scaffolds (Ghassemi et al., 2018). Other commercial products also have been manufactured for bone regeneragtion, including OSTEOSET^®^, OsSatura^®^, Cortoss^®^, Vitoss BA^®^,TruGraft™, ChronOS^®^, Healos^®^, and Calciresorb C35^®^ (Ge et al., 2008; Xue et al., 2022). In addition to their potency in delivering various factors that are required for cell survival and differentiation, scaffolds should have non-immunogenicity, non-cytotoxicity, and good biodegradability and biocompatibility properties (Dixon and Gomillion, 2021). Moreover, it has been shown that the vascularization system of scaffold plays a central role in the failure or success of bone repair by providing oxygen, minerals, growth factors, and nutrients, as well as discarding waste products from the regeneration microenvironment (Diomede et al., 2020). Thus, the development of scaffolds with available blood supply and coupling the angiogenesis with osteogenesis is a challenge in BTE. Hydrogels have attracted attention in BTE because of their good biocompatibility and porous structure, similar to the extracellular matrix (ECM). The soft texture of hydrogels also can minimize their inflammatory response in contact with adjacent cells and tissues (Buwalda et al., 2017). In this review, we summarized the requirements of BTE, the properties of hydrogels and their application in BTE, and the importance of angiogenesis during BTE. Finally, we focused on the effects of hydrogel-based scaffolds in promoting bone angiogenesis.

## 2 Bone tissue engineering and its requirements

Several processes and compartments play essential roles in bone regeneration and healing, including homing progenitor cells, osteoblast and osteocyte formation, ECM, and osteoid mineralization (Wang et al., 2013). The ECM is defined as a 3D and non-cellular structure containing specific polysaccharides and proteins secreted by cells into the extracellular space. Bone ECM comprises organic (collagen type I, glycoproteins, proteoglycans, small integrin-binding ligands N-linked glycoproteins, and γ-carboxyglutamate-containing proteins) and inorganic (hydroxyapatite) components (Lin et al., 2020). During bone defect healing, two main processes are involved: intramembranous ossification and endochondral ossification. Intramembranous ossification is associated with a direct transition of progenitor cells to bone-forming osteoblasts and forms the flat bones of the jaw and skull, whereas endochondral ossification is associated with a cartilage intermediate before the formation of bone and forms long bones (Bahney et al., 2019; Dewey and Harley, 2021). The BTE aims to prepare biomaterials to mimic ECM roles in mineralized matrix deposition and support cellular attachment after introducing them to the defect site. To this end, the compartments of BTE are reviewed in detail in the following sections.

### 2.1 Scaffolding materials

Scaffolds are temporary mechanical structures consisting of metals, ceramics, and polymers, in most cases, to provide an environment for ECM mimicking and bone remodeling with negligible complications (Kantaros et al., 2016). To design an ideal scaffold in BTE, four main types of requirements could be considered: biomaterial composition (ceramics, polymers, composites, etc.), structural features (bioinspired, customized shapes, biomimetic, surface topography, mechanical properties, pore interconnection, and high porosity), biological requirements (smart, bioactive, non-toxic, bioresorbable, biodegradable, non-immunogenic, and biocompatible), and fabrication process (conventional techniques: solvent casting, gas foaming, and freeze drying; and advanced techniques: rapid prototyping and electrospinning) (Roseti et al., 2017). The advantages and disadvantages of the most used scaffolds are listed in Table 1.

**TABLE 1:** The advantages and disadvantages of the most used scaffolds in BTE.

Scaffolds	Advantages	Disadvantages	Ref
Polymers	- Personalized manufacturing	- Slow biodegradability	Thavornyutikarn et al. (2014), Shi et al. (2016)
	- Good mechanical properties	- Inflammatory reactions because of acid degradation products	
	- Biocompatible		
	- High young modulus		
Ceramics	- Good mechanical properties	- Low elasticity	Seitz et al. (2005), Bose and Tarafder (2012), De Witte et al. (2018)
	- Personalized manufacturing	- Hard and brittle	
	- Resistance to corrosion		
	- Biocompatible		
Metals	- Biocompatible	- Bioinert	De Witte et al. (2018), Li Y et al. (2020)
	- Excellent mechanical properties	- Risk of toxicity with metal ions	
	- Personalized manufacturing	- Risk of corrosion	
	- Osteointegration	- Slow biodegradability	
Composites	- Supporting cell activity	- High stiffness	Gao C et al. (2014), Thavornyutikarn et al. (2014)
	- Excellent biocompatibility	- Low flexibility	
	- Osteoconductivity	- Brittle	
	- Easy to handle		

Among the structural features, the scaffold’s pore size and porosity are determining factors in the success of BTE due to their roles in the delivery of nutrients and vascularization. Although the high porosity of scaffolds guarantees the ingrowth of bone cells, their excessive porosity may negatively affect mechanical properties (Kuttor et al., 2014). There is evidence that pores with a size of almost 100 μm favor nutrient transport and cell migration (Zhang B. et al., 2018), whereas pores with ≥200 μm support vascularization and bone formation (Walpole et al., 2009). Studies also concluded that small pores (50–100 μm) were optimal for prompting endochondral ossification and large pores with 100–300 μm improved intramembranous ossification and enhanced vascularization (Wu et al., 2010; Daugela et al., 2018). In addition to pore size, grain size (structural dimension or the dimension of particles or grains within the scaffold and the size between the pores) of scaffolds also affects cellular adhesion, differentiation, and proliferation. For instance, Zhang et al. (2014) found that surface microstructural features of tricalcium phosphate (TCP) ceramics, TCP-S and TCP-B, have an indispensable role in their osteoinduction capacity. They reported that TCP-S (grain size < 1 μm) could stimulate osteogenic differentiation of human bone marrow stromal cells (BMSCs), characterized by overexpression of osteocalcin and osteopontin and increasing the activity of alkaline phosphatase (ALP) *in vitro*, compared with TCP-B with 3–4 μm grain size. After 12 weeks, the implantation of TCP-S into dog dorsal muscles led to the induction of bone formation, whereas TCP-B implants could not form any bone tissue. Furthermore, the optimal diameter for pore interconnectivity reported ranges from 700 to 1,200 μm, which supports the infiltration depth and bone deposition (Ghayor and Weber, 2018; Collins et al., 2021). Topography also could affect the osteogenic potential of progenitor cells within scaffolds. For instance, Zhou et al. (2016) constructed five nanorod-shaped 3D topographies with different interrod spacing and similar nanorod diameters and investigated their osteogenic effects on MSCs. They reported that interrod spacings <96 nm displayed dramatically enhanced osteogenic differentiation of MSCs, but constructs with interrod spacings >137 nm inhibited osteogenesis. Xu et al. (2017) found that fabricating islandlike structures on the nanofiber scaffold with chitosan provides appropriate interface for preosteoblast cell adhesion and proliferation and enhances their bone-forming capability. The stiffness is another property of scaffolds in determining the outcome of BTE. There is evidence that the stiffness of scaffolds affects cellular behavior in bone tissue, such as their proliferation, differentiation, migration, and contractility (Ping et al., 2021). In a study, Zhang J. et al. (2020) constructed 3D bone-like tissue constructs and evaluated the effects of scaffold stiffness on their osteogenesis potential. They indicated that soft scaffolds (0.66 kPa) not only induced osteogenic differentiation of stem cells and increased ALP activity, but also remarkably improved ECM mineralization and cellular organization compared with stiff scaffolds (5.4 kPa). On the other hand, Chatterjee et al. (2010) reported that the higher hydrogel stiffness could enhance osteogenic differentiation and later mineralization. Due to the conflicting reports, the effect of scaffold stiffness on osteogenic differentiation requires more investigation.

### 2.2 Cells

Although cell-free scaffolds have been applied in BTE, providing exogenous cells with osteogenesis potential is crucial for the damaged tissue lacking osteoprogenitor cells (Maisani et al., 2017). To this end, various cells have been used in BTE, including embryonic stem cells (ESCs), MSCs, dental stem cells, adipose-derived stem cells (ADSCs), and induced pluripotent stem cells (iPSCs).

ESCs, derived from the blastocyst’s inner cell mass (ICM), are pluripotent stem cells with the capability to differentiate into all cell types that teratoma formation *in vivo* and ethical restrictions are their concerns, whereas MSCs are considered “safe cells” for tissue regeneration (Lalu et al., 2012; Goradel et al., 2018). The osteogenic properties of MSCs could be related to their potential to generate a pro-osteogenic microenvironment through the secretion of paracrine factors. For instance, Ogata et al. (2017) revealed that cytokines in the secretomes of MSCs play a central role in osteoclastogenesis, as well as the recruitment and proliferation of angiogenic and osteogenic cells. Another study indicated that MSCs not only could improve bone regeneration and angiogenesis by producing various growth factors in a critical size calvarial defect, but also could suppress immune response initiation with paracrine effects (Gao X. et al., 2014). Moreover, scaffolds and their products also are involved in the osteogenic differentiation of MSCs. Naruphontjirakul et al. (2019) found that spherical monodispersed strontium containing bioactive nanoparticles (Sr-BGNPs) induced differentiation of MSCs into bone-forming cells and improved ECM mineralization. Mechanistically, Sr ions were the main actor in the osteogenic differentiation of MSCs without any cytotoxicity effects on their viability. ADSCs attracted increasing attention in bone regeneration due to their easy isolation and harvest and their proliferative and differentiative capacity into osteogenic and angiogenic linages (Paduano et al., 2017). For example, Calabrese et al. (2016) showed that combining human ADSCs (hADSCs) with collagen/hydroxyapatite scaffold promoted the differentiation of hADSCs into osteoblasts, characterized by ECM mineralization and maturation and upregulation of osteocalcin, osteopontin, and osterix. In another study, Jin et al. (2019) compared the osteogenic and angiogenic potential of ADSCs and dental pulp stem cells (DPSCs). They concluded that ADSCs showed higher osteogenic potential and upregulated osteoblast-related genes with superior mineral deposition, whereas DPSCs promoted angiogenesis with higher proliferative and migratory potential. Moreover, their implantation into a mandibular defect indicated that ADSCs could promote greater and faster bone regeneration in rats after 6 weeks.

### 2.3 Biochemical factors

To initiate the repair process, biochemical factors, in addition to scaffold and cells, are required to provide signals for attracting inflammatory and progenitor cells to the injured site. Three types of cues are required to induce osteogenesis: physical (mechanical action and morphology), chemical (composition differences and crosslinking), and biological. Among these cues, the physical factors are mentioned throughout the review; thus, we focus on chemical and biological factors here.

It has been shown that the mineral contents could affect the bone healing process. For instance, Zheng et al. (2014) found that β-TCP addition to the collagen solution showed notably better biological and mechanical properties in scaffolds. Mechanistically, the released Ca^2+^ from β-TCP activated calcium-sensitive receptors, leading to better newly formed bone in terms of quality and quantity without adverse effects. In another study, Wu et al. (2012) constructed cobalt (Co)-containing mesoporous bioactive glass (Co-MBG) scaffolds for mimicking hypoxia in BTE, in which Co acted as a chemical inducer of hypoxia-inducible factor 1α (HIF-1α). They reported that Co ions remarkably upregulated HIF-1α, vascular endothelial growth factor (VEGF), and bone-related genes in MSCs, and supported the proliferation and attachment of MSCs to the scaffold. In addition to the mineral contents, crosslinking is another chemical factor affecting BTE. There is evidence that collagen crosslinking plays a critical role in stimulating angiogenesis and stabilizing its conformation (Lindert et al., 2016). Kikuchi et al. (2004) indicated that adding glutaraldehyde, as a crosslinker, to hydroxyapatite/collagen (HA/Col) could improve the mechanical characteristics of the scaffold. Although glutaraldehyde can lead to adverse immune responses in the host system, its use in 8% is considered non-toxic and safe (Krishnakumar et al., 2019).

Various growth factors have been incorporated into the scaffolds to accelerate bone regeneration, including VEGF, bone morphogenetic proteins (BMPs), transforming growth factor β (TGFβ), insulin-like growth factor (IGF), and fibroblast growth factors (FGFs) (Azevedo and Pashkuleva, 2015). These growth factors are classified into inflammatory, osteogenic, and angiogenic. Since inflammation is the first step in healing bone fractures and inflammatory cells are recruited to the injured site, inflammatory factors are vital in BTE, such as macrophage colony-stimulating factor (M-CSF), interleukin-1 (IL-1), IL-6, and FGF2 (De Witte et al., 2018). Multiple pro-osteogenic growth factors have been used to recruit progenitor cells and promote their differentiation into bone-forming cells, including BMPs, IGF, FGF, TGFβ, and platelet-derived growth factor (PDGF) (Lienemann et al., 2012). The FDA has approved BMP-2 and BMP-7 incorporation in bone regeneration systems (Farokhi et al., 2016). Furthermore, IGF-1 acts as a mitogenic factor and is released when osteoclasts resorb fractured bone matrix, promoting the growth and differentiation of embryonic cells to osteoblasts (Kim et al., 2012; Nyberg et al., 2016). Angiogenic factors are discussed in detail in the following sections.

## 3 Hydrogel scaffolds in bone tissue engineering

**FIGURE 1:**
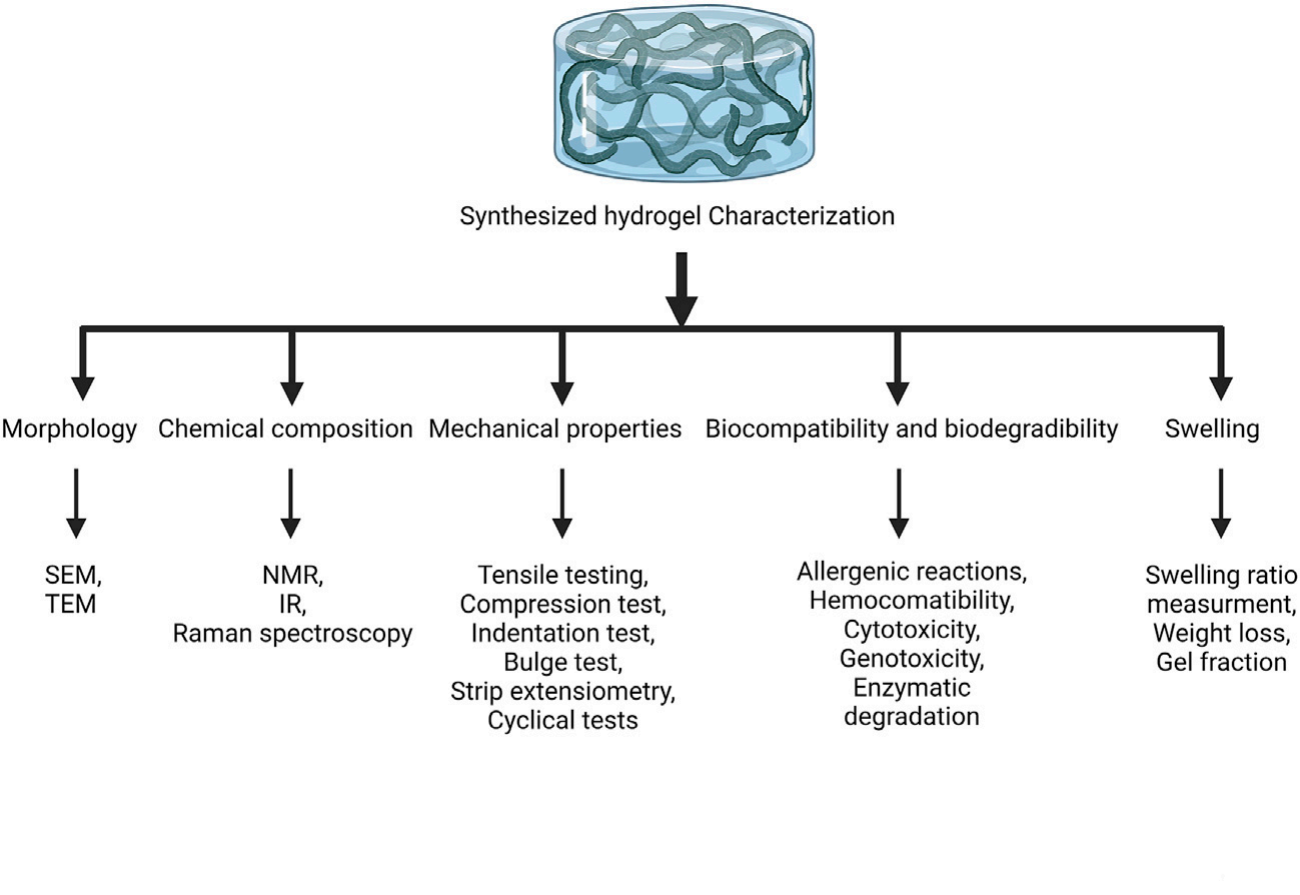
Characterization of hydrogels. The synthesized hydrogels are characterized by evaluating their morphology, chemical composition, mechanical properties, biocompatibility and biodegradability, and swelling. SEM, scanning electron microscope; TEM, transmission electron microscope; NMR, nuclear magnetic resonance; IR, infrared.

Hydrogels are 3D network structures with high water content (more than 90%) that are formed with crosslinking among hydrophilic polymers. Hydrogels promote cell survival, proliferation, and differentiation relying on providing a microenvironment similar to ECM in terms of mechanics and architecture. They are widely classified into two classes: natural hydrogels, which are derived from natural polymers, including collagen, hyaluronic acid (HA), alginate, chitosan, gelatin, and fibrin; and synthetic hydrogels, which are derived from chemically modified natural biopolymers or synthetic polymers (Zhu and Marchant, 2011). Although naturally derived hydrogels are attractive for BTE owing to their good biocompatibility, they suffer from the potential immunogenic response, uncontrollable biodegradation rate, low mechanical strength, low stiffness, and variability in production. The poor mechanical properties of hydrogels limit their application as load-bearing structures (Li J. et al., 2022; Charlet et al., 2022). On the other hand, synthetic hydrogels, such as polycaprolactone (PLC), polylactic acid (PLA), polyoxyethylene (PEO), poly (vinyl alcohol) (PVA), and poly (ethylene glycol) (PEG), possess more extended durability, tough mechanical strength, and flexible structure, while lacking biological activity (Xue et al., 2021). In addition to classification based on their origin, hydrogels are also classified based on their durability (durable and degradable), response to environmental stimuli (conventional and smart), charge (neutral, cationic, anionic, and ampholytic), structure (semi-crystalline and amorphous), and composition (homopolymer, copolymer, and semi-interpenetrating network) (El-Sherbiny and Yacoub, 2013). Various parameters have been determined to characterize hydrogels, including morphology, chemical composition, mechanical properties, biocompatibility and biodegradability, and swelling, which are evaluated by different techniques (Figure 1).

Hydrogels in BTE require some primary properties: compatibility with tissue and cells, osteoconductive activity, and osteoinductive activity. The ability of hydrogels to form new bone on their surface refers to osteoconduction, while the osteoinduction characteristic is related to the ability of hydrogels to absorb endogenous growth factors, induce the migration of stem cells into the scaffold, and promote bone formation (Yue et al., 2020). The development of hydrogels in BTE tries to cover the primary properties. For instance, Kazemi-Aghdam et al. (2021) introduced Icariin (IC)-loaded modified halloysite nanotubes (mHNTs) into chitosan hydrogel to develop a nanocomposite hydrogel for BTE. In the IC@mHNTs construct, IC acted as a bone inducer agent that promotes osteogenic differentiation. The *in vitro* studies demonstrated that the scaffold is a biocompatible structure that enhances the proliferation and differentiation of encapsulated MSCs. In another study, Saravanan et al. (2018) investigated the properties and bone regeneration capacity of thermosensitive chitosan (CS) and glycerophosphate (GP) based hydrogel containing graphene oxide (GO) (CS/GP/GO). They indicated that the CS/GP/GO hydrogel with pores >150 μm and ∼50% porosity was biocompatible to MSCs, promoting osteogenic differentiation of MSCs, characterized by overexpression of osteocalcin, ALP, Runt-related transcription factor 2 (Runx2), and Type -1 collagen. Also, the potential of carbon dots/hydroxyapatite/poly (vinyl alcohol) (CDs/HA/PVA) double-network (DN) hydrogel for BTE was reported by Liu et al. (2020) through the ability of the hydrogel to support MSCs proliferation and differentiation as well as repair bone defects *in vivo*. Evidence shows that the mechanical properties, degradation behavior, swelling behavior, and water content of CDs/HA/PVA DN hydrogel are determined by the number of freezing/thawing cycles (Wang Y. et al., 2019). Recently, Dibazar *et al.* fabricated a hydrogel nanocomposite containing bacterial polyglucuronic acid (PGU) and sodium alginate (Alg) composite with carbon nanofibers (CNFs) to study its bone regeneration properties. The hydrogel with interconnected pores architecture and biocompatible characteristics was hemocompatible with insignificant hemolysis induction. Furthermore, the PGU/Alg/CNFs hydrogel supported the growth of bone cells, suggesting it as a new scaffold for BTE. Table 2 summarizes the application of other hydrogels in BTE and their therapeutic effects on bone regeneration.

**TABLE 2:** The application of hydrogels in BTE.

Composition	Preparation	SC/GF	Characterization	Effects/results	Biodegradability (days)	Ref
CMCh-ACP hydrogel	pH-triggered, Self-assembled	BMP-9	DLS, SEM, TEM, FTIR, Viscosity, Injectability, pH responsiveness	Biocompatibility, Osteoinductive, Supporting MSC differentiation and adhesion, Enhancing bone formation	NA	Zhao et al. (2019)
GelMA-PEGDA-nHA composite hydrogel	Continuous ultrasound	—	SEM, FTIR, Swelling ratio, Degradation, Mechanical properties	Biocompatibility, Biodegradability, Supporting osteoblast adhesion and proliferation	56	Wang et al. (2020)
Electrospun nanofiber mesh and alginate hydrogel	Carbodiimide chemistry	rhBMP-2	2D radiographs and 3D *in vivo* μCT imaging, Histological analysis, Torsional testing	Enhancing bone formation, Improving infiltration of osteoprogenitor cells without adverse effects on revascularization	NA	Kolambkar et al. (2011)
GNF-collagen injectable hybrid hydrogel	Thermo-gelation process	hASCs	FTIR, Rheology analysis, SEM	Supporting cell adhesion and proliferation, Promoting differentiation of hASCs, Enhancing bone-like structures formation	NA	Maisani et al. (2018)
Oxidized alginate-gelatin hydrogel	Covalently crosslinked	mBMSCs	SEM, Porosity, FTIR, Degradation behavior	Supporting osteogenic differentiation of mBMSCs	28	Sarker et al. (2016)
GHH hydrogel	Enzyme-catalyzed	TMSCs	Blood analysis, visceral fat mass measurements, μCT Imaging	Biocompatibility, Reducing visceral fat, Enhancing bone formation	NA	Kim G et al. (2018)
GO-CS hybrid hydrogel	Crosslinking	hDPSCs	FTIR, XRD, SEM, EDX, Swelling tests, Weight loss evaluation	Enhancing minerals deposition, Supporting osteogenic differentiation of hDPSCs	28	Amiryaghoubi et al. (2020)
Cx-HA hydrogel	Mixing	BP/hDPSCs	Rheology analysis, Injection force, Fluorescence imaging	Biocompatibility, Promoting osteogenic differentiation of hDPSCs	28	Park et al. (2020)
Alginate/FmocFF composite hydrogel	Solvent switch	Preosteoblast cells	SEM, Rheology analysis	Biocompatibility, Facilitating calcium mineralization, Promoting osteogenic differentiation, Exhibiting excellent mechanical properties	NA	Ghosh et al. (2019)

SC, stem cell; GF, growth factor; CMCh, carboxymethyl chitosan; ACP, amorphous calcium phosphate; DLS, dynamic light scattering; SEM, scanning electron microscopy; TEM, transmission electron microscopy; FTIR, fourier transform infrared spectroscopy; MSC, mesenchymal stem cell; NA, not available; GelMA, gelatin methacrylamine; PEGDA, poly (ethylene glycol) diacrylate; nHA, nano hydroxyapatite; PECE, PEG-PCL-PEG, copolymer; rhBMP-2, recombinant bone morphogenetic protein-2; GNF, Glyco-nucleo-lipids containing a fluorinated carbon chain; hASCs, human adipose tissue-derived mesenchymal stromal cells; mBMSCs, murine bone marrow stromal cells; GHH, gelatin-hydroxyphenyl propionic acid; TMSCs, tonsil-derived mesenchymal stem cells; GO, graphene oxide; CS, chitosan; hDPSCs, human dental pulp stem cells; XRD, X-ray diffraction; EDX, energy dispersive X-ray; Cx, click-crosslinking; HA, hyaluronic acid; BP, BMP-2, mimetic peptide; FmocFF, fluorenylmethoxycarbonyl-diphenylalanine.

## 4 Importance of angiogenesis in bone development and repair

**FIGURE 2:**
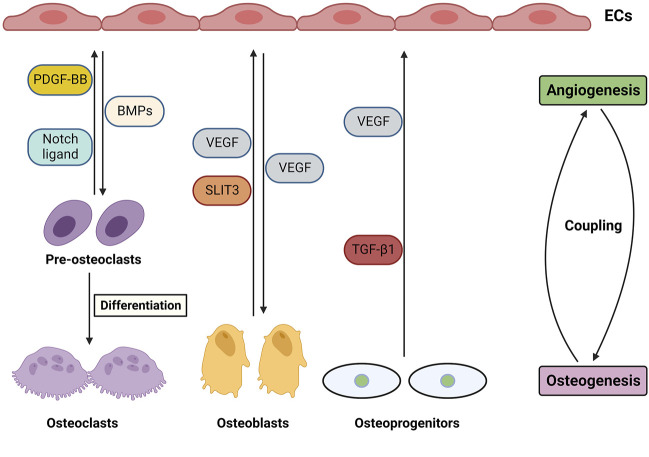
Angiogenesis and osteogenesis coupling in bones through cellular and molecular mediators and their crosstalk.

It has been reported that the vasculature system not only transports nutrients and oxygen and recruits cells within the bone, but also plays crucial roles in regulating osteogenesis and bone repair (Grosso et al., 2017; Di Maggio and Banfi, 2022). Therefore, fabricating scaffolds supplying functional blood is considered a challenge for BTE to couple osteogenesis and angiogenesis. Several factors and mediators regulate osteogenesis and type H vessel formation, including VEGF, Notch, HIF-1α, SLIT3, and PDGF-BB, which are produced by endothelial cells (ECs), osteoblasts, osteoclasts, and chondrocytes (Figure 2). The roles of factors are discussed below in detail.

### 4.1 VEGF

There is evidence that the VEGF family is the master regulator of angiogenesis. The VEGF family is composed of five members, including VEGF-A, -B, -C, -D, and placental growth factor (PlGF), and three cognate receptors, including VEGF-R1, -R2, and -R3 (Goradel et al., 2017; Hashemi Goradel et al., 2018). Although angiogenesis is a complex process, VEGF-A and VEGF-R2 are the key ligand and receptor involved in angiogenesis (Goradel et al., 2019). Various cell types in the bone microenvironment, such as recruited inflammatory cells and osteoprogenitors, express VEGFs that interact with their receptors on osteoclasts, pericytes, and again inflammatory cells and osteoprogenitors (Hu and Olsen, 2017).

Deleting VEGF and its receptors in osteoblasts and osteoblast precursors have been reported to impair their differentiation, leading to low bone density and bone development (Liu et al., 2012; Duan et al., 2015; Duan et al., 2016). Berendsen and Olsen (2014) indicated that VEGF directly controls the fate of mesenchymal stromal progenitor, in which VEGF blocks adipogenesis and stimulates osteoblastic differentiation. Interestingly, they found that the intracellular VEGF determines the fate of stem cells, not the exogenous one. Furthermore, the interaction between the VEGF family members with the ECM could regulate angiogenesis. Different affinity degrees of three major isoforms of VEGF-A transcript resulting from alternative splicing to ECM and the balance between their binding and diffusivity in physiological conditions plays an important role in controlling the osteogenic microenvironment (Gianni-Barrera et al., 2020; Di Maggio and Banfi, 2022). It is worth noting that type H vessels, which are characterized by higher expression of Endomucin (Emcn) and CD31, could induce osteogenesis by stimulating the proliferation and differentiation of osteoprogenitor cells (Peng et al., 2020).

### 4.2 Notch

The Notch receptors (Notch1–5) initiate signaling pathways after binding to their ligands, including Delta-like (Dll) 1, 3, or 4 and the Jagged family of Serrate homologs (Jag1, and Jag2), leading to cleavage and translocation of Notch intracellular domain (NICD) into the nucleus which coordinates cell proliferation, apoptosis, and differentiation (Pakvasa et al., 2021). It has been shown that the Notch signaling pathway plays dual roles in bone tissue development depending on cell type, the timing of signaling activation, and the stage of cell differentiation. The activation of Notch signaling promotes the differentiation of MSCs into osteoblasts and bone mineralization and suppresses osteogenesis *via* inhibiting the Wnt/β-catenin pathway (Luo et al., 2019). Recently, Kraus et al. (2022) showed that Notch signaling activation could accelerate cartilage callus conversion into bone and increase bone repair, whereas the inhibition of the signaling pathway delays the transformation of the cartilage callus into bone. In another study, Goel et al. (2019) reported that Notch signaling suppression in osteoclasts improves bone formation rate and healing in mice, characterized by larger bony calluses and increased bone mineral density. Additionally, blood flow in type H vessels regulates Notch signaling; a low rate suppresses the pathway, leading to defective osteogenesis and angiogenesis (Ramasamy et al., 2016). On the other hand, there is evidence that activation of Notch signaling could induce the formation of type H vessels (Tang et al., 2021). Liao et al. (2017) investigated the angiogenesis-osteogenesis coupling in MSCs in response to dual stimulation with BMP-9 and Notch signaling. They indicated that NICD1 promoted osteogenic differentiation of MSCs in a BMP-9-dependent manner. Furthermore, the implantation of transduced MSCs with BMP-9 and NICD1 in combination with a biocompatible scaffold induced bone formation with upregulation of angiogenic regulators and extensive vascularization.

### 4.3 HIF-1α

Under normoxic conditions, the hypoxia-inducible factor-1 α (HIF-1α) transcription factor undergoes the ubiquitin-proteasome degradation pathway following hydroxylation of the lysine and proline residues on the oxygen-dependent degradation domain and interacts with the Von Hippel–Lindau (VHL) E3 ubiquitin ligase (Pereira et al., 2006). To adapt cells with hypoxia conditions, the stable HIF-1α forms a heterodimer with HIF-1β, leading to the translocation of HIF-1α to the nucleus and activating the transcription of various target genes (Jin et al., 2020). There is evidence that HIF-1α is involved in both osteogenesis and angiogenesis and their coupling. Due to the hypoxic condition of the bone microenvironment in defects, Zhang J. et al. (2018) used preconditioned MSCs with hypoxia to repair bone. The hypoxic MSCs overexpressed HIF-1α, which enhanced the osteogenesis and angiogenesis potential of MSCs and improved their survival rate under severe conditions *in vitro*. The transplantation of hypoxic MSCs into critical-sized mandible defects in aged rats also improved bone defects. Also, the highly expressed HIF-1α MSCs, in combination with calcium-magnesium phosphate cement (CMPC) scaffold, repaired critical-sized calvarial defects in rats by promoting the upregulation of osteogenic markers (Zou et al., 2011). Due to the stabilizing effect of cobalt ions on HIF-1α, Quinlan et al. (2015) incorporated resorbable bioactive glass particles with cobalt ions into the glass network to fabricate bioactive glass/collagen–glycosaminoglycan scaffolds. They reported that the constructed scaffolds supported osteoblast proliferation and osteogenesis and promoted tubule formation. Recently, Amir et al. (2022) indicated that HIF-1α plays an indispensable role in BMP-9-mediated preosteoblasts differentiation into osteoblasts and vascularization, whereas HIF-1α knockdown or inhibition significantly suppressed the expression of osteogenic markers and matrix mineralization in a BMP-9-dependent manner. In another study, Guo et al. (2020) revealed that the protective activity of salidroside on bone loss is related to its stimulatory effects on angiogenesis-osteogenesis coupling *via* regulating the HIF-1α/VEGF axis.

### 4.4 SLIT3

Although slit guidance ligand 3 (SLIT3) is considered an axon-guidance molecule, there is evidence that it also plays physiological functions outside the nervous system, including carcinogenesis, regulation of stem cells, immunoregulation, and skeletal development (Li N. et al., 2020). Kim B.-J. et al. (2018) revealed that SLIT3 could stimulate the proliferation and migration of osteoblasts through the activation of β-catenin. It also inhibited the differentiation of osteoclast in an autocrine manner, resulting in suppression of bone resorption, whereas *Slit3* or its receptor, *Robo1*, deficient mice showed osteopenic phenotypes because of promoting bone resorption and inhibition of bone formation. Furthermore, higher levels of circulating SLIT3 were related to increased bone mass in postmenopausal women. In another study, Xu et al. (2018) tried to clarify how to type H vessels positively coordinate bone formation. They demonstrated that osteoblast-derived SLIT3 acts as a proangiogenic factor, promoting bone formation and increasing type H vessel numbers. On the other hand, *Slit3* deletion in osteoblast cells not only reduced skeletal type H vessels, but also decreased osteoblasts’ activity and reduced bone formation. Moreover, SLIT3-mutant mice exhibited defective fracture repair, whereas exogenous SLIT3 administration improved bone healing. These results suggest the essential roles of SLIT3 in coupling osteogenesis and angiogenesis.

### 4.5 PDGF-BB

Platelet-derived growth factor (PDGF) family consists of five members, including PDGF-AA, PDGF-AB, PDGF-BB, PDGF-CC, and PDGF-DD, in which PDGF-BB is a potent angiogenic, chemoattractant, and mitogenic molecule that is considered as a regulatory factor in tissue regeneration (Fredriksson et al., 2004; Wang C. et al., 2019). There is evidence that PDGF-BB can promote MSCs-based bone regeneration (Caplan and Correa, 2011; Zhang N. et al., 2021). In this regard, Zhang M. et al. (2018) investigated how PDGF-BB promotes MSCs-based bone regeneration by genetically modifying PDGF-BB-overexpressing MSCs. They exhibited that PDGF-BB not only suppressed adipogenic differentiation and enhanced osteogenic differentiation of MSCs, but also increased the migration of ECs and angiogenesis through regulation of the PI3K/AKT and ERK1/2 pathways. Also, PDGF-BB-overexpressing MSCs improved osteogenesis and angiogenesis during bone regeneration in a critical-sized rat calvarial defect model. Xie et al. (2014) found that the secreted PDGF-BB from preosteoclasts increased type H vessel numbers and bone formation during bone modeling remodeling. In contrast to the mentioned studies, Luvizuto et al. (2016) reported that the supplementation of biomaterials with PDGF-BB had no remarkable effects on bone formation. Despite the beneficial effects, PDGF-BB has a short half-life within the blood; thus, local and sustained delivery of PDGF-BB is essential to achieve ideal outcomes.

## 5 Hydrogel scaffolds promote bone angiogenesis

**FIGURE 3:**
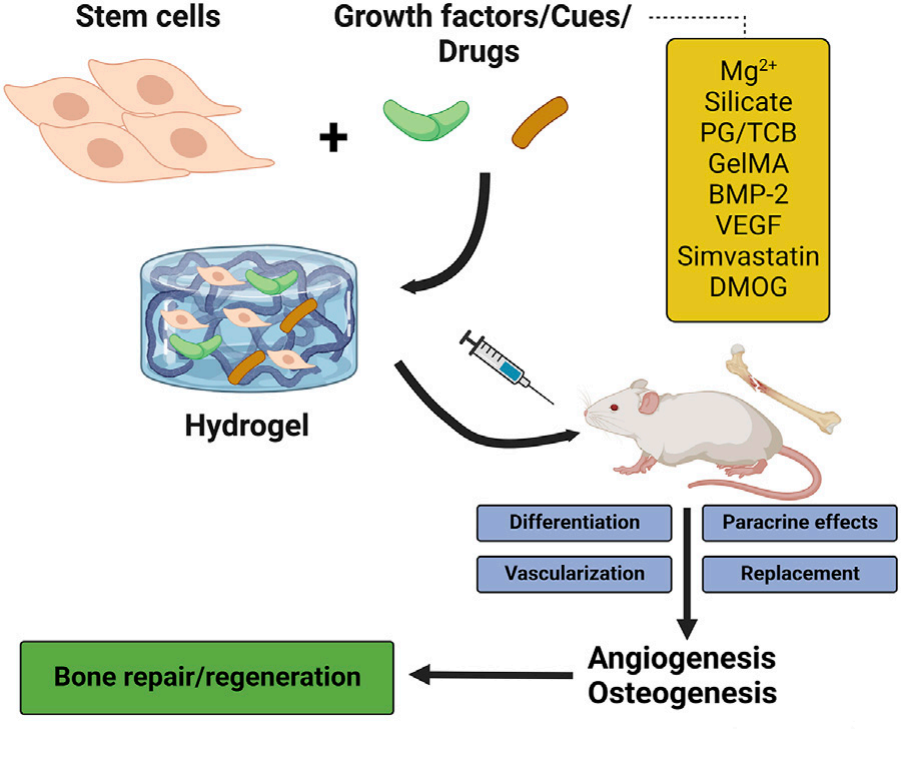
The stimulatory effects of hydrogels on osteogenesis and angiogenesis. PG/TCB, poly ethylene glycol maleate citrate (PEGMC) with β-TCP; GelMA, gelatin methacrylate; BMP-2, bone morphogenetic protein 2; VEGF, vascular endothelial growth factor; DMOG, dimethyloxallylglycine.

Before considering the importance of hydrogel scaffolds in providing structures for enhancing bone angiogenesis, it is worth noting that hydrogel effectively creates contiguous hyaline articular cartilage. Owing to the avascular structure of cartilage, its self-healing ability is limited during cartilage damage. On the other hand, biomaterials for the replacement of hyaline cartilage that lubricates joint movement exhibited undesirable side effects (Beddoes et al., 2016). To overcome these side effects, hydrogels have been introduced as promising material due to their excellent lubrication ability, producing contiguous hyaline articular cartilage matrix and seamless integration with native cartilage under the regular mechanical forces, constructing matchable structures with the different hyaline cartilage present in the body (Beddoes et al., 2016; Meppelink et al., 2016). Regarding the importance of angiogenesis during bone development and regeneration, fabricating hydrogel scaffolds with angiogenic properties or supporting systems for angiogenesis besides their osteogenesis capacity is an advantage for BTE (Figure 3). We divided harnessing hydrogel capcities in the activation of angiogenesis and bone regeneration into four strategies as below.

### 5.1 Ion-incorporated hydrogels

Due to the stimulatory effects of Mg^2+^ on cell differentiation and neovascularization in the bone microenvironment (Yoshizawa et al., 2014; Yu et al., 2017), Zhang X. et al. (2021) constructed an Mg^2+^-incorporating dual-crosslinked hydrogel using photopolymerization strategy and Mg-S coordination. The constructed hydrogel supported the adhesion, proliferation, spreading, and osteogenic differentiation of MSCs. The Mg^2+^-enriched hydrogel also enhanced the formation of tube-like structures and the number of branches in ECs, indicating its stimulatory effects on angiogenesis. The implantation of the Mg^2+^-enriched hydrogel into rats with a calvarial defect model improved bone formation and angiogenesis, characterized by upregulation of CD31 and higher density of blood vessels. Another ion that is incorporated into hydrogel scaffolds is the silicate ion due to its neovascularization capacity. Dashnyam et al. (2019) fabricated a silicate-shelled hydrogel fiber scaffold and examined its effect on bone regeneration. The released silicate and calcium ions from the scaffold upregulated angiogenic markers, including HIF1-α, bFGF, endothelial nitric oxide synthase (eNOS), KDR, and VEGF, and enhanced tubular networking in ECs. The implanted scaffold also improved bone formation and angiogenesis in rats. The released ions from the hydrogels could recruit progenitor cells to the bone microenvironment and stimulate the endogenous expression of VEGF, collectively resulting in bone repair (Yang et al., 2022). In another study, Sun et al. (2021) indicated that cobalt-incorporated hydrogels promote osteogenesis and angiogenesis. The tunable nanocomposite hydrogel with controlled release of cobalt ions induced osteogenesis and neovascularization and repaired the calvarial defect in a rat model because the released cobalt ion can mimic hypoxia to stimulate angiogenesis.

### 5.2 Vesicle-encapsulated hydrogels

In addition to the ions, MSC-derived vesicles also contribute to coupling osteogenesis and angiogenesis. For instance, exosomes affect cell-cell communication by carrying various materials and secret chemical signals (Zhang B. et al., 2021). It has been reported that pro-angiogenesis and pro-osteogenesis properties of MSCs-derived exosomes are associated with their effects on the activation of the HIF-1α/VEGF and the BMP-2/Smad1/Runx2 signaling pathways (Zhang L. et al., 2020). Recently, Wu D. et al. (2022) found that hydrogel-encapsulated small extracellular vesicles (sEVs) derived from MSCs with excellent thermosensitive properties could augment bone regeneration and repair calvarial defects *in vivo*. Mechanistically, MSCs-derived exosomes carry miR-21 that targets Sprouty2 (Spry2), leading to angiogenesis enhancement. Since Spry2 is an antagonist of fibroblast growth factor (FGF), there is evidence that Spry2 could control the integrity and quiescence of ECs and downregulate the angiogenesis process (Wietecha et al., 2011; Peier et al., 2013). Also, the pro-angiogenesis activity of exosomal miR-21 is associated with its modulatory effects on the NOTCH1/DLL4 pathway (Zhang Y. et al., 2021). In another study, Cheng et al. (2022) revealed that Nidogen1-enriched EVs (EV-NID1) enhanced the migration and angiogenesis potential of rat arterial endothelial cells (RAECs) by targeting myosin-10 which led to a reduction in the adhesion strength of RAECs. Moreover, loading EV-NID1 into a composite hydrogel accelerated angiogenesis and bone regeneration in an *in vivo* model of the femoral defect. NID1 is an ECM protein and an essential component of the vascular basement membrane that supports vascular and endothelium integrity (Marchand et al., 2019). Therefore, combining the delivery capacity of hydrogel with EVs with a high capacity to carry biological factors is a promising strategy to repair bone defects.

### 5.3 Hydrogels with antibacterial and immunomodulatory capabilities

**FIGURE 4:**
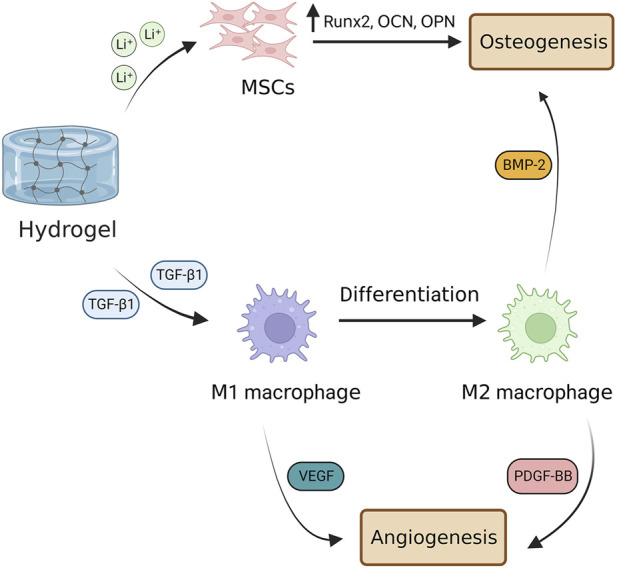
Hydrogel-based scaffolds enhance angiogenesis by modulating macrophage phenotype. Hydrogel-based scaffolds carry various ions and biomolecules, such as Li^+^ ions that promote differentiation of MSCs into bone-forming cells and TGF-β1 that stimulates M1 macrophage polarization toward M2 phenotype. M1 macrophages are involved in the early stage of bone angiogenesis by secreting VEGF, whereas M2 ones promote angiogenesis in the late stage by secreting PDGF-BB. M2 macrophage also is involved in osteogenesis through the secretion of BMP-2. TGF-β1, transforming growth factor beta 1; VEGF, vascular endothelial growth factor; PDGF-BB, platelet-derived growth factor-BB; BMP-2, bone morphogenetic protein 2.

Besides the osteogenesis and angiogenesis capacity, scaffolds need to be antibacterial with immunomodulatory capabilities. To this end, Cheng et al. (2020) constructed an injectable hydrogel with osteogenesis, angiogenesis, and antibacterial and called it ‘three-in-one’ platform, in which 4-arm-polyethylene glycol-thiol (4-arm-PEG-SH) hydrogel filled with liposomes-calcium phosphate nanoparticles (Lip#CaP). Firstly, they showed that the PEG-40Lip@DFO#CaP system is biocompatible owing to desirable effects on the viability of ECs. Furthermore, the treatment of ECs and preosteoblast cells with PEG-40Lip@DFO#CaP revealed the angiogenesis and osteogenesis potential of the hydrogel. The PEG-40Lip@DFO#CaP system also indicated antibacterial activities against *Escherichia coli, Staphylococcus epidermis, and Staphylococcus aureus* owing to carrying Ag^+^ and PEG in its structure. Lastly, they demonstrated that the implantation of the PEG-40Lip@DFO#CaP system into a rat calvarial critical-size defect model remarkably promoted osteogenesis and angiogenesis after 8 weeks. In another study, Ji et al. (2020) developed MSC-loaded thermosensitive hydroxypropyl chitin hydrogel (HPCH) incorporated with a 3D poly (ε-caprolactone) (PCL)/nano-hydroxyapatite (nHA) scaffold for BTE. The PCL/nHA + HPCH scaffold promoted the osteogenic differentiation of MSCs, characterized by the upregulation of osteopontin, osteocalcin, and Runx. Additionally, the PCL/nHA + HPCH scaffold enhanced angiogenesis by activating macrophages to secret VEGF and PDGF-BB. Furthermore, the MSCs-HPCH platform exerted immunomodulatory effects on macrophages by suppressing the M1 phenotype and their transition toward M2. Indeed, M1 macrophages secret VEGF and are involved in the initiation of angiogenesis; whereas M2 are responsible for the later stages of angiogenesis, stabilizing the vasculature, and typically secreting PDGF-BB. Consistent with *in vitro* results, the MSC-encapsulated PCL/nHA + HPCH hybrid scaffold enhanced bone formation and angiogenesis *in vivo*. Recently, Wu Z. et al. (2022) indicated that lithium (Li) -modified bioglass-hydrogel stimulates osteogenesis and neovascularization in a high glucose microenvironment by polarizing the differentiation of macrophage *in vitro*. Macrophage polarization from M1 toward M2 provided an anti-inflammatory microenvironment and relieved inflammation, resulting in bone regeneration in a diabetic rat bone defect. Similarly, Li D. et al. (2022) showed that incorporating TGF-β1 into Li-based hydrogel promotes both osteogenesis and angiogenesis, in which TGF-β1 guides macrophage polarization toward the M2 phenotype and Li ions enhance osteogenesis. Figure 4 represents how a hydrogel-based scaffold enhances angiogenesis by modulating macrophage phenotype.

### 5.4 Other strategies

Liu et al. (2019) developed a bioinspired 3D gelatin-methacrylate (Gel-MA) hydrogel incorporated with ECs and MSCs to study the capacity of the scaffold in bone vascularization. They reported that the co-culture of ECs and MSCs in the hydrogel system could promote angiogenesis both *in vitro* and *in vivo*. In this system, the differentiation of MSCs toward pericytes was the underlying mechanism of vascularization. In another study, Anada et al. (2019) prepared a bone-mimetic 3D hydrogel structure using GelMA, octacalcium phosphate (OCP), and ECs, and studied its osteogenesis-angiogenesis potential. The amount of OCP in the GelMA was a determinant factor in the proliferation and differentiation of MSCs; a higher amount of OCP decreased cell proliferation, while a higher amount of OCP increased othe steogenic differentiation of MSCs. Furthermore, GelMA concentration affects athe ngiogenesis and sprouting capacity of ECs; a higher GelMA concentration inhibits sprout formation. Chen et al. (2020) designed a gelatin-polyhedral oligomeric silsesquioxane (POSS) hybrid hydrogel (Gel-POSS) by an esterification reaction for BTE. They indicated that the introduction of POSS into gelatin provides a proper microenvironment for incubating ECs and MSCs on the hydrogel and supports angiogenic tube formation and extension. The addition of BMP-2 and VEGF to the hydrogel scaffold and its implantation into critical-sized rat calvarial defects revealed that the hydrogel supported the sustained release of the growth factors *in vivo*, leading to a higher blood vessel formation in defect regions. It has been shown that incorporating POSS moieties into biomaterials could enhance their matrix stiffness and make them porous structures (Wu et al., 2018; Tamburaci et al., 2019). Although simvastatin has a stimulatory effect on bone formation and simvastatin/poloxamer 407 hydrogel exhibited weak immunogenicity and low toxicity (Pillai and Panchagnula, 2003; Zhao et al., 2014; Tan et al., 2016), poor mechanical properties of thermosensitive simvastatin/hydrogel limit their application in BTE. On the other hand, although titanium alloys have low stiffness like cortical bone and are hopeful scaffolds for orthopedic uses, they are poorly compatible with bone growth owing to their bio-inert nature. Liu et al. (2016) designed 3D-printed porous titanium scaffolds (Ti_6_Al_4_V) containing simvastatin/hydrogel to use the advantages of both structures. They found that the constructed scaffolds not only significantly increased bone formation and bone mineral density, but also enhanced angiogenesis around and in the scaffolds. Dimethyloxallylglycine (DMOG) is another drug incorporated into the hydrogel scaffold in BTE. DMOG is an angiogenic drug that could improve both osteogenesis and vessel formation during bone repair. Mechanistically, the stimulatory effects of DMOG on osteogenesis and angiogenesis are due to HIF-1α activation (Yegappan et al., 2019). Therefore, incorporating pro-osteogenesis and pro-angiogenesis drugs into hydrogel scaffolds is a hopeful strategy to accelerate bone regeneration.

## 6 Conclusion

To date, various techniques have been developed to prepare hydrogels with desirable properties for BTE, including non-toxicity, good biocompatibility, controllability, and enhanced performance. Although more and more studies reported the advantages and benefits of hydrogels in BTE, the lack of clinical studies signifying their value limited their translation into the market. Some questions must be considered when designing polymers to construct hydrogels for BTE. First, will the hydrogel be used *in vitro* or *in vivo*? 2) Does the hydrogel constructed as 3D architecture or a space-filling one? 3) What is the fate of hydrogel in the long term? 4) Should cells be inert or be adhered to the hydrogel polymer? Currently, angiogenesis is a key bottleneck in BTE that is necessary to be overcome and considered. A thorough understanding of the bone repair phases to develop a reliable hydrogel-based scaffold is a required avenue for future studies.
